# Supplementary material for: Association between oral microbiome and seven types of cancers in East Asian population: a two-sample Mendelian randomization analysis
Source: Front Mol Biosci. 2023 Nov 21;10:1327893. doi: 10.3389/fmolb.2023.1327893 (PMC10702768; doi:10.3389/fmolb.2023.1327893)
Supplement: Supplementary file 1 [file DataSheet1.ZIP › Supplementary Materials/MR plots of saliva/saliva═╝/ovarian cancer/pheno.2182_to_ovarian cancer_forest.pdf]

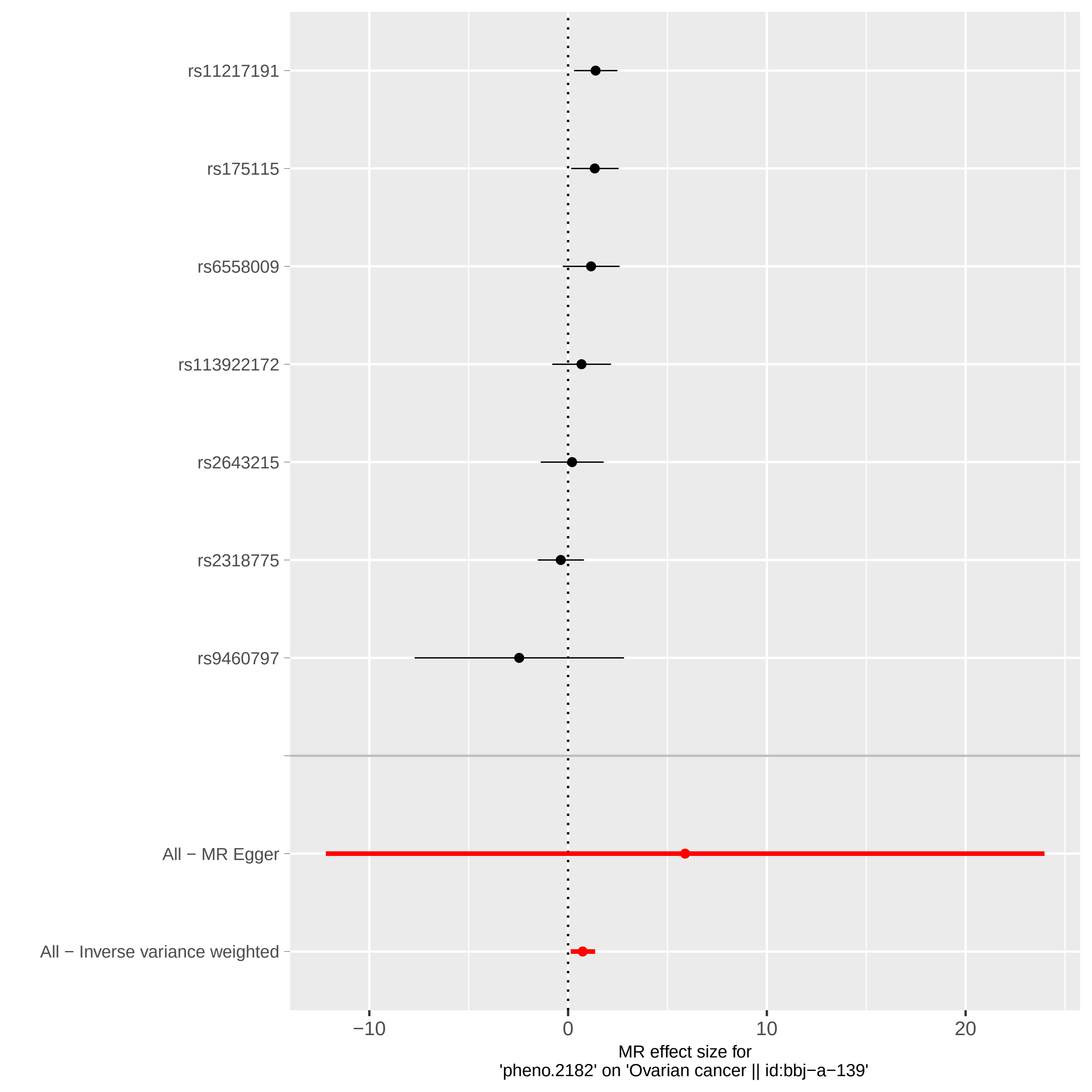

rs11217191
rs175115
rs6558009
rs113922172
rs2643215
rs2318775
rs9460797
All − MR Egger
All − Inverse variance weighted
−10
0
10
20
MR effect size for
'pheno.2182' on 'Ovarian cancer || id:bbj−a−139'
